# Urbanicity and rates of untreated psychotic disorders in three diverse settings in the Global South

**DOI:** 10.1017/S0033291722003749

**Published:** 2023-10

**Authors:** Tessa Roberts, Ezra Susser, Joni Lee Pow, Casswina Donald, Sujit John, Vijaya Raghavan, Olatunde Ayinde, Bola Olley, Georgina Miguel Esponda, Joseph Lam, Robin M. Murray, Alex Cohen, Helen A. Weiss, Gerard Hutchinson, Rangaswamy Thara, Oye Gureje, Jonathan Burns, INTREPID Group, Adejoke Agboola, Kulandaiyesu Amaldoss, Jothi Ramadoss Aynkaran, Abirami Balashanmugam, Darielle Bharath-Khan, Premalatha Chockalingam, Kruthika Devanathan, Olawoye Fadahunsi, Subhashini Gopal, Olufemi Idowu, Donella Jadoo, Triplicane Chakravarthy Ramesh Kumar, Elysse Marcellin, Clement Obuene, Akin Ojagbemi, Bamise Olayiwola, Seyi Owoeye, Padmavati Ramachandran, Elena Raymond, Karthick Samikannu, Grace Sooknanan, Lauren Subnaik, Diana Williams, Craig Morgan

**Affiliations:** 1 ESRC Centre for Society & Mental Health, Institute of Psychiatry, Psychology and Neuroscience, King's College London, London, UK; 2 Health Service and Population Research Department, Institute of Psychiatry, Psychology & Neuroscience, King's College London, London, UK; 3 Department of Epidemiology, Mailman School of Public Health, Columbia University, New York, USA; 4 New York State Psychiatric Institute, New York, USA; 5 Department of Psychiatry, University of the West Indies, St Augustine Campus, Trinidad & Tobago; 6 Schizophrenia Research Foundation, Chennai, India; 7 Department of Psychiatry, College of Medicine, University of Ibadan, Ibadan, Nigeria; 8 Department of Population, Practice and Policy, UCL Great Ormond Street Institute of Child Health, London, UK; 9 Division of Psychological Medicine, Institute of Psychiatry, Psychology & Neuroscience, King's College London, London, UK; 10 Department of Population Health, Faculty of Epidemiology and Population Health, London School of Hygiene & Tropical Medicine, London, UK; 11 MRC International Statistics & Epidemiology Group, Faculty of Epidemiology and Population Health, London School of Hygiene & Tropical Medicine, London, UK; 12 Mental Health Research Group, College of Medicine and Health, University of Exeter, Exeter, UK; 13 National Institute for Health Research, Mental Health Biomedical Research Centre at South London and Maudsley NHS Foundation Trust and King's College London, London, UK

**Keywords:** Epidemiology, global mental health, incidence, psychosis, schizophrenia, urbanicity

## Abstract

**Background:**

Extensive evidence indicates that rates of psychotic disorder are elevated in more urban compared with less urban areas, but this evidence largely originates from Northern Europe. It is unclear whether the same association holds globally. This study examined the association between urban residence and rates of psychotic disorder in catchment areas in India (Kancheepuram, Tamil Nadu), Nigeria (Ibadan, Oyo), and Northern Trinidad.

**Methods:**

Comprehensive case detection systems were developed based on extensive pilot work to identify individuals aged 18–64 with previously untreated psychotic disorders residing in each catchment area (May 2018–April/May/July 2020). Area of residence and basic demographic details were collected for eligible cases. We compared rates of psychotic disorder in the more *v.* less urban administrative areas within each catchment area, based on all cases detected, and repeated these analyses while restricting to recent onset cases (<2 years/<5 years).

**Results:**

We found evidence of higher overall rates of psychosis in more urban areas within the Trinidadian catchment area (IRR: 3.24, 95% CI 2.68–3.91), an inverse association in the Nigerian catchment area (IRR: 0.68, 95% CI 0.51–0.91) and no association in the Indian catchment area (IRR: 1.18, 95% CI 0.93–1.52). When restricting to recent onset cases, we found a modest positive association in the Indian catchment area.

**Conclusions:**

This study suggests that urbanicity is associated with higher rates of psychotic disorder in some but not all contexts outside of Northern Europe. Future studies should test candidate mechanisms that may underlie the associations observed, such as exposure to violence.

## Background

The incidence of psychotic disorders is approximately twice as high among people living in urban *v.* non-urban settings (Vassos, Pedersen, Murray, Collier, & Lewis, 2012), based on data from Northern Europe where this finding has been repeatedly replicated (March et al., 2008; Plana-Ripoll, Pedersen, & McGrath, 2018). Longitudinal studies examining residence in early life before the onset of psychosis (March et al., 2008) suggest that this finding cannot be explained solely by social drift (Pedersen, 2015) – in other words, the hypothesis that people who are vulnerable to psychotic disorders tend to migrate to more urban areas, or are unable to move out of them – and there is evidence of a cumulative effect of exposure to urban environment during childhood (Pedersen & Mortensen, 2001). Since the proportion of the global population that lives in urban settings is rapidly increasing, and 68% of the world's people are projected to live in cities by 2050 according to UN projections (United Nations, 2018), it is imperative that we understand the mechanisms underlying the association between urbanicity and psychosis, particularly in countries that are set to account for a large proportion of this growth, such as India and Nigeria (Robertson, 2019), and identify modifiable risk factors to avert corresponding increases in psychotic disorders.

The mechanisms underlying the association between urban living and psychosis are subject to ongoing debate. There is evidence of higher rates of psychosis among people belonging to ethnic minority or migrant groups in Northern Europe (Morgan, Charalambides, Hutchinson, & Murray, 2010), and the proportion of people from minority groups is typically higher in urban areas, but the urbanicity effect has been shown to persist in studies that have accounted for ethnicity (Richardson, Hameed, Perez, Jones, & Kirkbride, 2018). Candidate environmental risk factors include aspects of the physical environment, such as exposure to air pollution, infections, toxins and nutritional deficiency in utero (Attademo, Bernardini, Garinella, & Compton, 2017; March et al., 2008); and aspects of the social environment such as reduced social cohesion, increased neighbourhood disorder, greater inequality and increased exposure to crime victimisation have also been proposed to explain the excess risk (Kirkbride, Jones, Ullrich, & Coid, 2014; Newbury et al., 2016). Some studies have also suggested that gene-environment correlations could contribute (Grech & van Os, 2017; Sariaslan et al., 2015, 2016), due to genetic selection of which families live in urban areas.

However, the generalisability of the association between urban living and psychosis is unclear. The EU-GEI programme found evidence of this urbanicity effect only in Northern but not Southern Europe (Jongsma et al., 2018). There are very limited data on the incidence of psychotic disorders from the rest of the world (Bastien et al., 2021) and considerable variation in rates of psychosis between settings (Jongsma, Turner, Kirkbride, & Jones, 2019). Recent findings from the INTREPID II programme also demonstrate wide variation in rates when comparing settings in India, Nigeria and Trinidad (Morgan et al., 2022) but to date within-setting variation in incidence rates by level of urbanicity have not been explored. The evidence that is available on urbanicity and risk of psychosis from the global south suggests heterogeneity in this association across contexts (Kirkbride, Keyes, & Susser, 2018). For example, in the World Health Organization's Ten Country study, higher rates of schizophrenia were reported in Chandigarh (rural India) than in Agra (urban India) (Jablensky et al., 1992). More recently, two studies of the incidence of psychotic disorder from Brazil and Chile found no evidence of an association between the incidence of psychotic disorders and urbanicity, when operationalised in terms of population density (Del-Ben et al., 2019; González-Valderrama et al., 2020). In China the best available evidence comes from prevalence studies, with some studies finding no association of psychotic disorders with urban living while others have reported higher rates of psychosis in rural areas (Huang et al., 2019; Phillips et al., 2009).

Understanding the extent to which urban living is a context-specific risk factor for psychotic disorders, and why it may be present in some settings but not others, can help us to better understand which aspects of city life contribute to the increased risk, with the potential to inform preventative public health interventions. In this study, we therefore aimed to examine the association between urban residence and incidence of psychotic disorders in catchment areas of three diverse countries in the global south; India, Nigeria, and Trinidad.

## Methods

### Settings

The INTREPID II catchment areas comprise three economically, socially, and culturally diverse settings on three continents, and have been previously described elsewhere (Morgan et al., 2022).

In India, the catchment area comprises four taluks (administrative sub-districts) in Kancheepuram district, Tamil Nadu, to the South of Chennai city; Chengelpettu, Thiruporur, Uthiramerur and Maduranthakam, with a total population of 997 492 residents. Two of these taluks (Chengelpettu and Thiruporur) were previously combined and were split after the time of the last census, so for the purposes of the current analyses are treated as a single administrative area since population data are not yet available separately. Uthiramerur and Maduranthakam are predominantly rural, while Chengelpettu/Thiruporur can be considered predominantly urban (see Table 1; over half of its residents live in urban areas according to the binary census classification, described below, and only 14% of main workers in this area work in agriculture, compared with 61% and 52% in Uthiramerur and Maduranthakam, respectively).

**Table 1.** Selected indicators from the 2011 Census of India, 2006 Census of Nigeria, 2011 Census of Trinidad & Tobago and Central Statistical Office of Trinidad & Tobago archives (those relevant to urbanicity that are available at the level of local administrative areas)

	Population density (per sq km)	% population living in urban areas as per	% literate	% main workers agricultural labours/cultivators	% secondary education	% tertiary education	Average monthly household consumption expenditure (TT $)	% seeking work	% households with 1 or more people in work	% households renting/leasing (inc rent-free)	% rented govt housing	Immigration rate^a^ (total)	Serious crimes reported per 1000 population^b^	Violent crimes reported per 1000 population^c^
Kancheepuram (Tamil Nadu, India)														
Uthiramerur	737	17%	66%	61%	–	–	–	–	–	–	–	–	–	–
Maduranthakam	347	19%	67%	52%	–	–	–	–	–	–	–	–	–	–
Chengelpettu/Thiroporur	361	54%	76%	14%	–	–	–	–	–	–	–	–	–	–
Trinidad														
Port of Spain	2910	–	–	–	70%	18%	6878	4%	61%	42%	15%	3.4	52	31
Arima	2784	–	–	–	66%	22%	7458	4%	74%	19%	0%	2.5	27	19
Chaguanas	1415	–	–	–	66%	19%	6496	3%	75%	20%	0%	2.9	13	8
Diego Martin	812	–	–	–	70%	21%	8733	4%	67%	22%	1%	4.2	8	5
San Juan/Laventille	657	–	–	–	64%	14%	7160	4%	72%	32%	3%	2.8	7	5
Tunapuna/Piarco	417	–	–	–	66%	20%	7718	3%	66%	23%	23%	2.9	10	7
Sangre Grande	82	–	–	–	56%	11%	6809	4%	70%	12%	0%	1.6	10	8
Ibadan (Oyo, Nigeria)														
Ibadan NE	18 356	–	–	–	54%	–	–	–	–	–	–	–	–	–
Ibadan SE	15 650	–	–	–	50%	–	–	–	–	–	–	–	–	–
Ona-Ara	914	–	–	–	46%	–	–	–	–	–	–	–	–	–

a Units unclear – not specified by CSO.

b Serious crime includes murder, attempted murder, assault, rape, sexual assault and other sexual offences (including incest), serious indecency, kidnapping, burglary, robbery, larceny, fraud, narcotic offences, possession of firearms/ammunition, manslaughter, attempted suicide, malicious damage, arson, perverting the course of justice, misbehaviour in public office, etc. (Note that original CSO definition includes ‘etc’; no definitive list available).

c Violent crime includes murder, assault, rape, sexual assault and other sexual offences, kidnapping, burglary, robbery, possession of firearms and ammunition. (Classification created by the researchers for the purposes of the current study, and constrained by the original categories used for reporting).

In Nigeria, the catchment area includes three local government areas in Oyo State with a total population of 861 504, of which two are within the city of Ibadan – Ibadan North East and Ibadan South East – and a further local government area (Ona-Ara) is located on the periphery of the city and includes both urban and rural areas. Ona-Ara is partly agrarian, with farming populations scattered over its wide expanse of land. Other economic activities include textile weaving, dyeing and wood carving. Ibadan North-East and Ibadan South-East are commercial centres with large markets as well as banking and hospitality facilities. The only relevant census data available at the level of local government areas was educational attainment (Table 1).

In Trinidad, the catchment area comprises seven municipalities in the North of the island (Port of Spain, Arima, Chaguanas, Tunapuna/Piarco, San Juan/Laventille, Diego Martin and Sangre Grande), with a total population 705 296, spanning both urban and rural areas. Crime rates, education levels, noise exposure and the proportion of households in rented accommodation vary by municipality as shown in Table 1.

### Inclusion criteria and recruitment

As previously described (Roberts et al., 2020), we aimed to identify all individuals with a previously untreated psychotic disorder within each catchment area. ‘Untreated’ was defined for the purposes of the current study as having never received treatment with anti-psychotic medication for one continuous month prior to the start of the case-finding period. Additional inclusion criteria were being aged 18–64 years old, currently resident in the catchment area, meeting criteria for a diagnosis of psychotic disorder as per ICD-10 criteria (including affective, non-affective and substance-induced psychoses: F20, F22, F25, F30-31 (with psychotic features), F32-33 (with psychotic features), F10-19 (with psychotic features), F23, F28-29). Participants were excluded if they experienced only transient psychotic symptoms resulting from the acute intoxication, if they had moderate or severe learning disabilities, or if they had a clinically manifest organic cerebral disorder.

Our case-finding procedures were based on those developed in the INTREPID I pilot study (Morgan et al., 2015, 2016), and have been described in detail elsewhere (Morgan et al., 2022). In brief, we established case detection systems by mapping service provides and key informants in each catchment area, including the professional sector (mental health services), the folk sector (traditional and religious providers), and the popular sector (informal sources of support within the community). Researchers in each setting conducted regular checks with each provider and key informant – including checking clinical notes and registers where available – to identify individuals with a potential psychotic disorder, supported by materials based on qualitative work from INTREPID I on local terms used to describe the symptoms of psychosis (Cohen et al., 2016). Case-finding started on 1 May 2018 in all settings, and concluded 25 months later in the Indian setting, 27 months later in the Nigerian setting, and 24 months later in the Trinidadian setting (the total period of case detection varied according to the local situation in terms of coronavirus disease 2019 (COVID-19) restrictions to allow the research team to recontact each main provider/key informant before the end of the study, and we adjusted for the variable duration of case detection by setting when calculating person-years at risk). The Screening Schedule for Psychosis (Jablensky et al., 1992) was used to screen potential cases, which was followed by a clinical interview by a trained researcher using the Schedules for Clinical Assessment in Neuropsychiatry (SCAN) (Wing et al., 1990), with diagnoses assigned by a local psychiatrist based on the information collected from the SCAN. Where it was not possible to interview eligible individuals, the clinical diagnosis was recorded based on medical records. This was a small minority of cases in India and Nigeria but in Trinidad we identified far more cases than we were able to interview so we relied on clinical notes for 361/573 eligible cases. For those who we were able to interview, there were no disagreements between the research team and the treating clinicians as to whether an individual met criteria for a psychotic disorder, although there were some disagreements over the exact diagnosis.

Age, gender, area of residence and estimated duration of untreated psychosis (i.e. time from onset of psychotic disorder to the time of presentation to services or identification by the INTREPID II research team) were recorded for all eligible participants. Leakage studies were conducted in all settings at the end of the case-finding period (Morgan et al., 2022) and all cases who were confirmed to be eligible through this process were included in the total count for the purposes of estimating rates of untreated psychotic disorder.

### Variable definition and statistical analysis

**Fig. 1.**
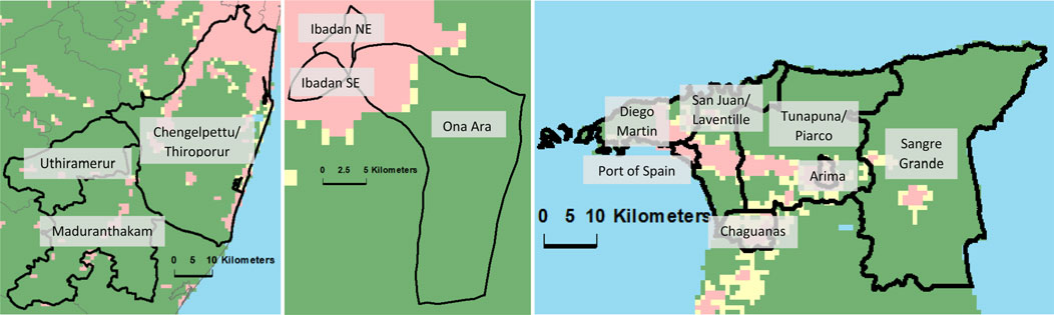
(*a*–*c*). Maps of INTREPID II catchment areas in India, Nigeria and Trinidad. Black borders indicate the boundaries of the total catchment area in each setting and the administrative areas within these. Green, yellow and pink voxels indicate level of urbanicity according to the GHS-SMOD classification system using the Degree of Urbanisation (DEGURBA) methodology, developed by EuroSAT (Florczyk et al., 2019; Joint Research Centre (JRC) European Commission, & Center for International Earth Science Information Network – CIESIN – Columbia University, 2021). EuroSAT's DEGURBA methodology. This applies a set of decision rules that consider population and built-up area densities derived from the GHS-POP and GHS-BUILT data sets, which use spatial data mining technologies that rely on a combination of fine-scale satellite image data streams, census data, and crowd sourced or volunteered geographic information sources (see https://sedac.ciesin.columbia.edu/data/set/ghsl-population-built-up-estimates-degree-urban-smod). Pink indicates urban areas (Class 30: ‘Urban Centre’, Class 23: ‘Dense Urban Cluster’, and Class 22: ‘Semi-dense Urban Cluster’), yellow indicates peri-urban areas (Class 21: ‘Suburban or peri-urban’), while green indicates rural areas (Class 13: ‘Rural cluster’, Class 12: ‘Low Density Rural’, and Class 11: ‘Very low density rural’).

We grouped the administrative areas within each setting into more and less urban areas, based on data from GHS-SMOD classification system using the Degree of Urbanisation (DEGURBA) methodology, developed by EuroSAT, which incorporates both population density and density of built up areas (Florczyk et al., 2019; Joint Research Centre (JRC) European Commission, & Center for International Earth Science Information Network – CIESIN – Columbia University, 2021). This provides a standard approach across the three settings (Fig. 1).

For current purposes we examine the effect of relative urbanicity within each setting, since urban living may indicate very different contexts of risk across diverse settings.

For comparison, in the Census of India urban areas were defined as ‘all places with a municipality, corporation, cantonment board or notified town area committee, etc. (‘Statutory Towns’), and all other places which satisfied the following criteria (‘Census Towns’); minimum population of 5000; at least 75% of the male main workers engaged in non-agricultural pursuits; and population density of at least 400 per sq. km’ (Census of India, 2011). In Trinidad, in the 2008/09 Household Budget Survey, wards with 200 or more residents per square kilometre were classified as urban and the remainder rural, with the exception of wards ‘located in urban areas with 40 or more agricultural holders and/or at least 48 hectares under agricultural cultivation (as reported in the 2004 Agricultural Census) with an element of remoteness such as distance from main cities or difficult access’, which were classified as rural (Central Statistical Office of Trinidad & Tobago, 2009). In Nigeria, an area is classified as urban on the basis of the population size (20 000 or more) or its administrative status (capital of a state or headquarter of a local government area).

Rates of untreated psychoses were calculated using Poisson regression at the level of administrative areas using Stata software (version 15) for Windows. To calculate the denominator, population data are taken from the most recent census in each country, which was 2006 in Nigeria, and 2011 in India and Trinidad, adjusted for projected population growth following the methods used in previous studies (Morgan et al., 2022). The population statistics used to calculate rates of psychosis are included in the supplementary material. These rates were adjusted for gender and age, using the direct standardisation method to the world population as reference (https://esa.un.org/unpd/wpp/Download/Standard/Population/) to enable comparisons across settings. We then calculated incidence rate ratios comparing more urban with less urban areas within each setting, using negative binomial regression (due to over-dispersion of the data) controlling for age and gender. It was not possible to control for other potential confounders due to the limitations of the data available. We also conducted sensitivity analyses by repeating this process while including only those with an onset of psychosis within the past 5 and the past 2 years, to assess the extent to which operationalising incidence in differing ways affected our findings.

### Ethics

This study was approved by the ethical review boards of King's College London (reference number: HR-17/18-5601), London, UK; London School of Hygiene and Tropical Medicine (reference number: 15807), SCARF, Chennai, India; the University of Ibadan, Ibadan, Nigeria; the University of the West Indies, St Augustine, Trinidad; and the North West, North Central, and Eastern Regional Health Authorities of Trinidad.

## Results

268, 196, and 574 eligible cases were identified in the Indian, Nigerian, and Trinidadian catchment areas, respectively. Residence data was missing for one case in Trinidad.

Table 2 shows the sample characteristics by local area [between-setting variations are reported elsewhere (Morgan et al., 2022)]. There was little variation in the gender distribution of the sample by local area in Trinidad or Nigeria. In India the proportion of men varied from 34.4% in Uthiramerur to 50.7% in Maduranthakam (both predominantly rural areas; in the more urban area of Chengelpettu/Thiroporur the proportion was 40.6%). Age at detection did not vary substantially by local area in any of the three settings. There was a larger proportion with an unspecified diagnosis in the Indian setting (41.8%) than the other two settings (8.7% in the Nigerian setting, 17.9% in the Trinidadian setting), but within-site variation in diagnoses was limited in all settings and there was no clear association of diagnostic category with urbanicity in any of the three settings.

**Table 2.** Distribution of age, sex and diagnostic group among incident cases, by local area

	Men, *n* (%)	Age at detection, median (IQR)	Non-affective diagnosis, *n* (%)	Affective diagnosis, *n* (%)	Psychosis NOS, *n* (%)
Kancheepuram (Tamil Nadu, India)	**114 (42.5)**	**42 (33–50)**	**147 (54.9)**	**9 (3.4)**	**112 (41.8)**
Uthiramerur	11 (34.4)	41.5 (30–50)	15 (46.9)	1 (3.1)	16 (50.0)
Maduranthakam	36 (50.7)	44 (35–50)	42 (59.2)	2 (2.8)	27 (38.0)
Chengelpettu/Thiroporur	67 (40.6)	42 (33–50)	90 (54.6)	6 (3.6)	69 (41.8)
Trinidad	**339 (59.1)**	**30 (23–40)**	**380 (66.2)**	**144 (25.1)**	**50 (8.7)**
Port of Spain	46 (63.0)	28 (21–36)	55 (75.3)	13 (17.8)	5 (6.9)
Arima	37 (51.4)	32 (23–43)	52 (56.3)	30 (29.1)	15 (14.6)
Chaguanas	46 (59.7)	33 (26–40)	48 (62.3)	20 (26.0)	9 (11.7)
Diego Martin	40 (59.7)	29 (22–39)	50 (74.6)	17 (25.4)	0 (0.0)
San Juan/Laventille	55 (56.1)	30 (23–38)	60 (61.2)	28 (28.6)	10 (10.2)
Tunapuna/Piarco	62 (60.2)	32 (24–42)	58 (56.3)	30 (29.1)	15 (14.6)
Sangre Grande	52 (62.7)	29 (23–36)	57 (68.7)	20 (24.1)	6 (7.2)
Ibadan (Oyo, Nigeria)	**103 (52.6)**	**34 (26–41)**	**125 (63.8)**	**36 (18.4)**	**35 (17.9)**
Ibadan NE	30 (54.6)	34 (26–41)	40 (72.7)	6 (10.9)	9 (16.4)
Ibadan SE	35 (53.0)	35.5 (27–45)	36 (54.6)	18 (27.3)	12 (18.2)
Ona-Ara	38 (50.7)	34 (25–40)	49 (65.3)	12 (16.0)	14 (18.7)

Values in bold refer to the values for the whole setting, whereas values not in bold are for the local administrative areas within these settings.

**Fig. 2.**
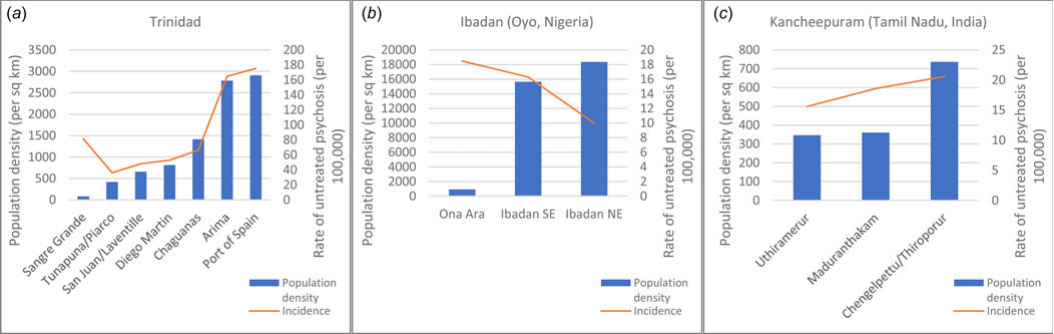
(*a*–*c*). Rate of psychosis and population density by area.

Table 3 shows the number of cases identified in each area and rates of untreated psychotic disorder. Figure 2(a–c) also shows population density and rates of psychotic disorder (adjusted for age and sex) in each of the three settings.

**Table 3.** Rate of untreated psychosis by area of residence (grouped into more and less urban areas) – all psychoses

Local area	Type of area	Pop density (per sq km)	Cases (all psychoses)	Crude rate^a^	Adjusted rate^b^ (95% CI)	IRR^b^ (within site)
Uthiramerur & Maduranthakam	Rural	356	103	16.6	17.6 (14.1–21.0)	1
Chengelpettu/Thiroporur	Peri-urban/mixed	737	165	19.6	20.6 (17.4–23.8)	1.18 (0.91–1.53)
Ona Ara	Peri-urban/mixed	914	75	17.9	18.5 (14.1–22.9)	1
Ibadan North-East & South-East	Urban	17 041	121	12.3	12.8 (10.4–15.1)	0.69 (0.50–0.96)
Sangre Grande, Tunapuna/Piarco, San Juan/Laventille, Diego Martin, Chaguanas	Mixed rural/peri-urban/urban	351	428	50.2	51.5 (46.6–56.4)	1
Arima & Port of Spain	Urban	3030	145	157.7	169.5 (141.6–197.4)	3.19 (2.54–4.00)

a Crude and adjusted rates are per 100 000 people.

b Adjusted for age and gender.

In Trinidad there is strong evidence of a positive association between population density and rates of psychosis, with more than a threefold increase in rates of psychosis in the most urban areas compared with the less urban areas. When we repeated the analysis while restricting to recent onset cases, similar findings were observed (<5 years: IRR 2.95, 95% CI 2.27–3.84; <2 years: IRR 2.55, 95% CI 1.93–3.36).

In the Indian setting, there was no evidence of variation in rates between more and less urban areas when considering all cases (IRR 1.18, 95% CI 0.91–1.53). When analyses were restricted to recent onset cases only, however, there was an association between urbanicity and increased rates of psychosis (5 years: IRR 1.66, 95% CI 1.15–2.42; <2 years: IRR 1.82, 95% CI 1.11–2.98).

In the Nigerian setting we observed lower rates in the more urban areas, both when we included all cases and when we restricted analyses to those with a recent onset only (overall IRR 0.69, 95% CI 0.50–0.96; <5 years IRR 0.63, 95% CI 0.44–0.89; <2 years IRR 0.54, 95% CI 0.36–0.81).

The supplementary material provides the incidence rate ratios by individual area, for comparison. The observed trends were the same across all three diagnostic sub-groups (non-affective, affective, and unspecified) (see supplementary material; note that we only conducted analyses by diagnostic sub-group when using the grouped areas due to the sparsity of data when simultaneously stratifying by both diagnosis and individual area).

## Discussion

This study represents the first analysis of urbanicity and risk of psychotic disorder to include multiple settings in the global south with comprehensive case-finding methods. Although our results should be interpreted with caution due to the limitations of the data, they represent an important first step in diversifying research on the link between urban living and psychosis beyond Europe and North America. We found evidence of a strong positive association between urbanicity and rates of psychosis in Trinidad, with threefold higher rates in the most urban areas compared with less urban areas. In India rates of psychosis were higher in more urban areas when excluding those with a long duration of untreated illness, but not without this exclusion. In Nigeria, it seems unlikely that there are higher rates in more urban areas as this was not observed in either the overall analysis or the sensitivity analyses. We observed the opposite relationship, with lower rates in the more urban areas, but caution is needed in interpreting these results as this may be a methodological artefact (discussed below).

These findings tentatively suggest that the association between urban residence and increased risk of psychotic disorder may be context-specific. Our findings most resembled previous results from Northern Europe in Trinidad, which is now classified as a high-income country (while Nigeria and India are both middle-income); the extent to which country income level is relevant to the urbanicity phenomenon is unclear, however. These results reinforce the need to identify the factors that contribute to increased risk in some – but not all – urban contexts.

Population density is only one characteristic of urban areas and the idea of crowding is unlikely to fully capture the experience of living in a city or in the countryside. Previous research has suggested that social cohesion may be a contributing factor to local variation in risk, and smaller settlements may be more cohesive (Heinz, Deserno, & Reininghaus, 2013; Kirkbride et al., 2007; Zammit et al., 2010). In Trinidad there is little evidence of increased migration in the more densely-populated municipalities, but there does appear to be a higher proportion of people living in rented accommodation, particularly government rentals, in Port of Spain, which may potentially indicate looser social ties to the neighbourhood. Furthermore, rates of serious crime are substantially higher in the more urban areas. Increased exposure to violence is a plausible candidate explanation that is hypothesised by the socio-developmental model of psychosis (Morgan et al., 2010), and may be linked with lower levels of social cohesion. Noise exposure – which might affect psychosis risk by disrupting sleep (Freeman, Sheaves, Waite, Harvey, & Harrison, 2020), interrupting learning (Lercher, Evans, Meis, & Kofler, 2002), or generating chronic low-level stress (Link, Dohrenwend, & Skodol, 1987) – is also more common in more urban settings in Trinidad. Finally, deprivation and inequality have been hypothesised to increase risk of psychosis (Burns & Esterhuizen, 2008; Kirkbride et al., 2014). In Trinidad there was no obvious association between population density and indicators of deprivation at the municipality level, but this may obscure important differences in relative poverty within municipalities, which may contribute to elevated rates in cities.

### Limitations

There are several limitations to this analysis. The first is that it included a small number of local areas, particularly in India and Nigeria, which limits the extent to which we can draw firm conclusions about urbanicity and rates of psychosis in these contexts. We also had to use a crude approach to classifying urbanicity as a dichotomous variable at the level of administrative areas, some of which included both rural and urban areas; had the data allowed, a more nuanced approach (e.g. Dahly & Adair, 2007) might have detected associations that were obscured by our strategy of grouping areas into two broad categories. In particular, Ona Ara in Nigeria includes both rural and urban areas, so it is possible that relatively high rates of psychosis in the urban section of this local government area drove the unexpected finding of higher rates compared with Ibadan North East and South East.

The second limitation relates to uncertainty in the numerator used to calculate rates: It is possible that we systematically missed more cases in some areas than others (e.g. if people in rural areas are less likely to seek formal care and are therefore harder to find, or if urban cases are more likely to travel out of the catchment area for care). In Ibadan, the most densely-populated area included in the study, case-finding was particularly challenging within the city because it has a larger number of small care providers operating outside of the formal health system. It is therefore possible that we missed more cases in the urban Nigerian setting than in the mixed/peri-urban area, contributing to the finding of lower than expected rates of psychosis within the city. Case-finding is likely to have been most comprehensive in Trinidad, where cases were almost exclusively identified through public mental health services which are free and catchment-area based. We can therefore be most confident of our findings in the Trinidadian setting, which do suggest an increased risk of psychosis in more urban areas. Nonetheless, our reliance on care providers for case detection in this setting does mean that we may have missed cases who were homeless, confined by their families, or who sought treatment abroad, and it is theoretically plausible that this could differentially affect case-finding in more *v.* less urban areas. It is also possible that services are more accessible in urban areas, leading to an under-estimation of rural rates of psychosis.

Thirdly, there is some uncertainty in the denominator. In all settings the underlying population at risk was estimated based on census data from several years prior to the study, which introduces some uncertainty into estimates. This is particularly problematic in Nigeria, where the last census took place in 2006. New censuses are due in all three settings but have been delayed due to the COVID-19 pandemic.

Fourthly, the exposure and outcome were measured simultaneously. These analyses are based on participants' current area of residence, rather than where they grew up, which in some cases will differ. Evidence from Northern Europe suggests that exposure during childhood and adolescence may be the most relevant to the onset of psychotic symptoms (Pedersen & Mortensen, 2001). Given the cross-sectional nature of these data we cannot rule out social drift as an explanation for the higher rates of psychosis in more urban areas in the Trinidadian and Indian catchment areas (data on migration were collected for those who went on to participate in other components of the INTREPID II study but it was not possible to collect these for all eligible cases identified: these data are limited but suggest relatively low levels of migration in all catchment areas). Conversely, it is possible that we did not observe higher rates in more urban areas in Nigeria because the cultural norm is to send relatives with mental illness out to the countryside to recover, either due to stigma or to aid recovery. Qualitative research may help to investigate this possibility.

Finally, we did not control for family history of psychosis, and some (although not all) previous studies have found that the association between urbanicity and psychosis risk disappears after accounting for this (Maxwell, Coleman, Breen, & Vassos, 2021; Sariaslan et al., 2015, 2016). It therefore remains possible that higher rates in more urban areas of Trinidad are attributable to familial selection factors, although given the effect sizes reported it seems unlikely that this could account for differences of the magnitude reported in Trinidad.

### Implications and recommendations for future research

This study provides the first preliminary evidence that urbanicity is associated with increased risk of psychosis in Trinidad, although social drift cannot be entirely ruled out as an explanation. This suggests a potential need to invest in more high-quality services for people with psychosis within cities in Trinidad, where rates are exceptionally high. This study adds to the previous finding of high rates in Trinidad (Morgan et al., 2022) by showing large within-setting variation in incidence, with an adjusted rate in the most urban areas of 169.5 per 100 000 [for comparison, the highest rate reported in a recent systematic review was 90.0 per 100 000 (Jongsma et al., 2019)]. This points to an extremely high burden of need within urban Trinidad, and an urgent need to identify the processes responsible for driving such high rates. The last incidence study to be conducted in Trinidad reported vastly lower rates of psychosis (Bhugra et al., 1996). One major contextual change that has occurred between these two time points is the transformation of Trinidad into one of the most violent settings in the world as a result of shifts in the international narcotics trade, which has led to an exponential increase in gang-related crime and an influx of illicit substances and firearms (Knight, 2019). Port of Spain (the capital) has been most affected by drug trafficking and violent crime (Baird, Bishop, & Kerrigan, 2022). This speculative hypothesis for the exceedingly high incidence of psychosis in urban Trinidad can be examined in upcoming analyses of INTREPID II data on exposure to violence and drug use among cases and controls.

In Tamil Nadu (India), findings are more tentative, since risk of psychosis was associated with urbanicity only after excluding cases with a long duration of untreated psychosis. Indeed, the most pressing need in this context might be to invest more resources into earlier identification of people with psychoses in rural areas. In Nigeria, by contrast, our results suggest that is very unlikely that the risk of psychosis is higher in more urban areas, although the limitations of case-finding in urban areas do not allow us to entirely rule this out.

It is imperative to identify the specific factors that account for the elevated rates observed in urban areas in many settings. Known risk factors such as cannabis use and exposure to childhood trauma may be more common among urban dwellers (although we found little evidence of substantive differences in the prevalence of cannabis use by area, see supplementary material). There may also be differences in deprivation, social fragmentation and income inequality between urban and rural areas, all of which have been implicated in neighbourhood effects in incidence of psychosis in Northern Europe (March et al., 2008), although in Trinidad socio-economic variables do not appear to be closely correlated with population density at the municipality level. Air pollution is also more common in cities, which has been linked to mental health including psychosis (Attademo et al., 2017; King, Zhang, & Cohen, 2022; Newbury et al., 2019), but this would predict higher rates of mental health problems in large cities in the global south than the global north, where there are fewer public transport options and air quality is worse. Hypotheses about exposure to violence, social fragmentation and excessive noise should be tested in future studies, with large enough samples, sufficiently disaggregated data, and within sufficiently heterogeneous contexts to assess their relative contributions to explaining variations in psychosis rates. To test hypotheses about social processes such as social cohesion or fragmentation, locally-valid measures are needed to capture these variables across diverse settings. Longitudinal studies in more diverse contexts (i.e. including the global south) are also needed to identify critical periods for exposure and to rule out social drift.

## Conclusion

This study found preliminary evidence of substantially increased rates of psychotic disorders in more urban areas of Trinidad compared with less urban areas. This association was not observed in Ibadan (Oyo, Nigeria) and was observed to a smaller degree and only when restricting to recent onset cases in Kancheepuram (Tamil Nadu, India). These findings should be interpreted tentatively in light of the limitations of the available data, and require replication before basing policy and service planning on these results. Further studies are needed to test candidate mechanisms underlying this association and identify the characteristics of some urban settings that could be targeted to prevent psychosis.

## Supplementary material

For supplementary material accompanying this paper visit https://doi.org/10.1017/S0033291722003749. click here to view supplementary material
