# Supplementary material for: Urbanicity and rates of untreated psychotic disorders in three diverse settings in the Global South
Source: Psychol Med. 2023 Jan 16;53(14):6459–67. doi: 10.1017/S0033291722003749 (PMC10600928; doi:10.1017/S0033291722003749)
Supplement: Supplementary file 1 [file S0033291722003749sup001.docx]

**Supplementary material**

**Table 3b.** Rate of untreated psychosis by area of residence (grouped into more and less urban areas) – non-affective diagnoses only

| **Local area** | **Type of area** | **Pop density**  **(per sq km)** | **Cases (non-affective)** | **Crude**  **rate*** | **Adjusted rate** (95% CI)** | **IRR** (within site)** |
| --- | --- | --- | --- | --- | --- | --- |
| Uthiramerur & Maduranthakam | Rural | 356 | 57 | 9.2 | 9.8 (7.2-12.3) | 1 |
| Chengelpettu/Thiroporur | Peri-urban/ mixed | 737 | 90 | 10.7 | 11.3 (8.9-13.6) | 1.16 (0.81-1.68) |
| Ona Ara | Peri-urban/ mixed | 914 | 49 | 11.7 | 11.8 (8.3-15.2) | 1 |
| Ibadan North-East & South-East | Urban | 17041 | 76 | 7.7 | 8.1 (6.2-10.0) | 0.66 (0.46-0.95) |
| Sangre Grande, Tunapuna/Piarco, San Juan/ Laventille, Diego Martin, Chaguanas | Mixed rural/ peri-urban/ urban | 351 | 273 | 32.0 | 33.0 (29.1-26.9) | 1 |
| Arima & Port of Spain | Urban | 3030 | 107 | 116.4 | 123.9 (100.2-147.6) | 3.79 (2.89-4.98) |

*Crude and adjusted rates are per 100,000 people
**Adjusted for age and gender

**Table 3c.** Rate of untreated psychosis by area of residence (grouped into more and less urban areas) – affective diagnoses only

| **Local area** | **Type of area** | **Pop density**  **(per sq km)** | **Cases (affective)** | **Crude**  **rate*** | **Adjusted rate** (95% CI)** | **IRR** (within site)** |
| --- | --- | --- | --- | --- | --- | --- |
| Uthiramerur & Maduranthakam | Rural | 356 | 3 | 0.5 | 0.5 (0.0-1.0) | 1 |
| Chengelpettu/Thiroporur | Peri-urban/ mixed | 737 | 6 | 0.7 | 0.7 (0.1-1.3) | 1.48 (0.37-5.91) |
| Ona Ara | Peri-urban/ mixed | 914 | 12 | 2.9 | 3.4 (1.3-5.5) | 1 |
| Ibadan North-East & South-East | Urban | 17041 | 24 | 2.4 | 2.3 (1.4-3.3) | 0.86 (0.40-1.87) |
| Sangre Grande, Tunapuna/Piarco, San Juan/ Laventille, Diego Martin, Chaguanas | Mixed rural/ peri-urban/ urban | 351 | 115 | 13.5 | 13.6 (11.1-16.1) | 1 |
| Arima & Port of Spain | Urban | 3030 | 29 | 31.5 | 34.2 (21.6-46.8) | 2.37 (1.58-3.57) |

*Crude and adjusted rates are per 100,000 people
**Adjusted for age and gender

**Table 3d.** Rate of untreated psychosis by area of residence (grouped into more and less urban areas) – psychosis NOS only

| **Local area** | **Type of area** | **Pop density**  **(per sq km)** | **Cases (NOS)** | **Crude**  **rate*** | **Adjusted rate** (95% CI)** | **IRR** (within site)** |
| --- | --- | --- | --- | --- | --- | --- |
| Uthiramerur & Maduranthakam | Rural | 356 | 43 | 6.9 | 7.3 (5.1-9.5) | 1 |
| Chengelpettu/Thiroporur | Peri-urban/ mixed | 737 | 69 | 8.2 | 8.6 (6.6-10.6) | 1.19 (0.81-1.74) |
| Ona Ara | Peri-urban/ mixed | 914 | 14 | 3.3 | 3.3 (1.5-5.1) | 1 |
| Ibadan North-East & South-East | Urban | 17041 | 21 | 2.1 | 2.3 (1.3-3.4) | 0.63 (0.32-1.24) |
| Sangre Grande, Tunapuna/Piarco, San Juan/ Laventille, Diego Martin, Chaguanas | Mixed rural/ peri-urban/ urban | 351 | 40 | 4.7 | 4.9 (3.4-6.4) | 1 |
| Arima & Port of Spain | Urban | 3030 | 9 | 9.8 | 11.4 (3.9-19.0) | 2.15 (1.02-4.54) |

*Crude and adjusted rates are per 100,000 people
**Adjusted for age and gender

**Table 4.** Population and population density by area

| **Country** | **Administrative area** | **Total population** | **Population at risk (age 18-64)** | **Sq km** | **Pop density (per sq km)** |
| --- | --- | --- | --- | --- | --- |
| Nigeria | Ibadan NE | 330399 | 245,353 | 18 | 18356 |
|  | Ibadan SE | 266046 | 193,331 | 17 | 15650 |
|  | Ona-Ara | 265059 | 186,305 | 290 | 914 |
| Trinidad | Port of Spain | 34,914 | 23,274 | 12 | 2910 |
|  | Arima | 33,404 | 22,705 | 12 | 2784 |
|  | Chaguanas | 83,498 | 58,937 | 59 | 1415 |
|  | Diego Martin | 102,340 | 66,841 | 126 | 812 |
|  | San Juan/Laventille | 157,021 | 104,254 | 239 | 657 |
|  | Tunapuna-Piarco | 212,825 | 145,902 | 510 | 417 |
|  | Sangre Grande | 75,605 | 50,332 | 927 | 82 |
| India | Chengelpettu (includes Thiruporur) | 573,406 | 403,359 | 747 | 737 |
|  | Uthiramerur | 148,133 | 104,203 | 347 | 347 |
|  | Maduranthakam | 275,953 | 194,118 | 764 | 361 |

**Table 5.** Rate of untreated psychosis by area of residence (individual municipalities/taluks/local government areas)

| **Local area** | **Pop density**  **(per sq km)** | **Cases (all psychoses)** | **Crude**  **rate*** | **Adjusted rate** (95% CI)** | **Spearman’s Rho (p-value)** | **IRR****  **(within site)** |
| --- | --- | --- | --- | --- | --- | --- |
| Uthiramerur | 347 | 32 | 14.7 | 15.6 (10.1-21.0) | - | 1 |
| Maduranthakam | 361 | 71 | 17.6 | 18.6 (14.3-23.0) |  | 1.19 (0.78-1.82) |
| Chengelpettu/ Thiroporur | 737 | 165 | 19.6 | 20.6 (17.4-23.8) |  | 1.33 (0.90-1.96) |
| Ona Ara | 914 | 75 | 17.9 | 18.5 (14.1-22.9) | - | 1 |
| Ibadan  South-East | 15650 | 66 | 15.2 | 16.3 (12.2-20.4) |  | 0.85 (0.59-1.23) |
| Ibadan  North-East | 18356 | 55 | 10.0 | 10.0 (7.3-12.8) |  | 0.56 (0.38-0.82) |
| Sangre Grande | 82 | 83 | 82.5 | 81.5 (63.9-99.1) | 0.61 (0.15) | 1 |
| Tunapuna/ Piarco | 417 | 103 | 35.3 | 36.3 (29.2-43.3) |  | 0.45 (0.33-0.61) |
| San Juan/ Laventille | 657 | 98 | 47 | 48.4 (38.8-58.1) |  | 0.58 (0.43-0.80) |
| Diego Martin | 812 | 67 | 50.1 | 53.1 (40.3-65.9) |  | 0.64 (0.46-0.90) |
| Chaguanas | 1415 | 77 | 65.3 | 66.3 (51.4-81.1) |  | 0.82 (0.59-1.13) |
| Arima | 2784 | 72 | 158.5 | 164.9 (126.5-203.2) |  | 2.03 (1.46-2.83) |
| Port of Spain | 2910 | 73 | 156.8 | 175.4 (134.7-216.0) |  | 2.07 (1.49-2.88) |

*Crude and adjusted rates are per 100,000 people
** Adjusted for age and gender

**Table 6.** Putative risk factors among controls by administrative area

The following data were collected from age- and gender-matched controls (as a proxy for general population data) as part of the INTREPID II study. Methods of control recruitment are described in Roberts et al (2020). Cannabis use was measured using the Alcohol, Smoking and Substance Involvement Screening Test (ASSIST) (Humeniuk et al, 2008).

|  | **Type of area** | **N** | **Population density (per sq km)** | **Lifetime cannabis use (%)** | **p-value** | **Problematic cannabis use* (%)** | **p-value** |
| --- | --- | --- | --- | --- | --- | --- | --- |
| Uthiramerur & Maduranthakam | Rural | 87 | 356 | 1.15 | 0.21 | 1.15 | 0.21 |
| Chengelpettu/Thiroporur | Peri-urban/ mixed | 138 | 737 | 0.0 |  | 0.0 |  |
| Ona Ara | Peri-urban/ mixed | 53 | 914 | 11.3 | 0.87 | 5.7 | 0.91 |
| Ibadan North-East & South-East | Urban | 77 | 17041 | 10.4 |  | 5.2 |  |
| Arima & Port of Spain | Mixed rural/ peri-urban/ urban | 56 | 3030 | 67.9 | 0.47 | 21.8 | 0.14 |
| Sangre Grande, Tunapuna/Piarco, San Juan/Laventille, Diego Martin, Chaguanas | Urban | 156 | 351 | 62.8 |  | 32.5 |  |

*Defined as ASSIST score of >3 (“moderate involvement” or “high involvement”, for which the WHO recommends intervention)
Missing substance use data for 3 participants in Trinidad (2 from mixed area, 1 from urban area). Those with missing data were excluded from the analysis

**Tables 7-10.** Sensitivity analyses when restricting to recent-onset cases (<2 years/<5 years) – grouped and by individual area

Table 7. Rate of untreated psychosis by area of residence (<5 years duration only – by individual area)

| **Local area** | **Pop density**  **(per sq km)** | **New cases (5yrs)** | **Crude**  **rate*** | **Adjusted rate** (95% CI)** | **IRR** (within site)** |
| --- | --- | --- | --- | --- | --- |
| Uthiramerur | 347 | 13 | 6.0 | 6.4 (2.9-9.9) | 1 |
| Maduranthakam | 361 | 27 | 6.7 | 6.7 (4.1-9.2) | 1.11 (0.58-2.16) |
| Chengelpettu/ Thiroporur | 737 | 90 | 10.7 | 11.1 (8.8-13.4) | 1.78 (1.00-3.20) |
| Ona Ara | 914 | 65 | 15.5 | 16.0 (11.9-20.1) | 1 |
| Ibadan South-East | 15650 | 49 | 11.3 | 11.9 (8.4-15.4) | 0.72 (0.48-1.09) |
| Ibadan North-East | 18356 | 47 | 8.5 | 8.3 (5.8-10.8) | 0.54 (0.40-0.83) |
| Sangre Grande | 82 | 69 | 68.5 | 67.3 (51.4-83.2) | 1 |
| Tunapuna/ Piarco | 417 | 78 | 26.7 | 27.7 (21.5-33.9) | 0.41 (0.29-0.58) |
| San Juan/ Laventille | 657 | 80 | 38.4 | 39.5 (30.8-48.2) | 0.58 (0.41-0.82) |
| Diego Martin | 812 | 61 | 45.6 | 48.1 (35.9-60.2) | 0.72 (0.50-1.03) |
| Chaguanas | 1415 | 63 | 53.4 | 54.8 (41.2-68.4) | 0.82 (0.57-1.18) |
| Arima | 2784 | 51 | 112.3 | 117.7 (85.1-150.2) | 1.75 (1.19-2.56) |
| Port of Spain | 2910 | 60 | 128.9 | 146.4 (109.0-183.8) | 2.08 (1.44-3.00) |

**Table 8.** Rate of untreated psychosis by area of residence (<2 years duration only – by individual area)

| **Local area** | **Pop density**  **(per sq km)** | **New cases (2yrs)** | **Crude**  **rate*** | **Adjusted rate** (95% CI)** | **IRR** (within site)** |
| --- | --- | --- | --- | --- | --- |
| Uthiramerur | 347 | 7 | 3.2 | 3.2 (0.8-5.6) | 1 |
| Maduranthakam | 361 | 15 | 3.7 | 3.6 (1.8-5.5) | 1.15 (0.47-2.82) |
| Chengelpettu/ Thiroporur | 737 | 54 | 6.4 | 6.6 (4.8-8.4) | 1.99 (0.91-4.38) |
| Ona Ara | 914 | 47 | 11.2 | 11.8 (8.2-15.4) | 1 |
| Ibadan South-East | 15650 | 34 | 7.8 | 8.2 (5.3-11.2) | 0.68 (0.43-1.08) |
| Ibadan North-East | 18356 | 27 | 4.9 | 4.6 (2.8-6.4) | 0.43 (0.26-0.70) |
| Sangre Grande | 82 | 62 | 61.6 | 60.7 (45.5-75.8) | 1 |
| Tunapuna/ Piarco | 417 | 68 | 23.3 | 24.1 (18.4-29.9) | 0.39 (0.28-0.56) |
| San Juan/ Laventille | 657 | 70 | 33.6 | 34.7 (26.6-42.9) | 0.56 (0.40-0.79) |
| Diego Martin | 812 | 59 | 44.1 | 46.7 (34.7-58.6) | 0.76 (0.53-1.09) |
| Chaguanas | 1415 | 59 | 50.1 | 51.4 (38.2-64.6) | 0.84 (0.59-1.20) |
| Arima | 2784 | 40 | 88.1 | 93.2 (64.1-122.3) | 1.50 (1.01-2.24) |
| Port of Spain | 2910 | 48 | 103.1 | 116.2 (83.0-149.5) | 1.83 (1.25-2.66) |

**Table 9.** Rate of untreated psychosis by area of residence (<5 years duration only – grouped)

| **Local area** | **Type of area** | **Pop density**  **(per sq km)** | **New cases (5yrs)** | **Crude**  **rate*** | **Adjusted rate** (95% CI)** | **IRR** (within site)** |
| --- | --- | --- | --- | --- | --- | --- |
| Uthiramerur & Maduranthakam | Rural | 356 | 40 | 6.4 | 6.6 (4.5-8.6) | 1 |
| Chengelpettu/  Thiroporur | Peri-urban/ mixed | 737 | 90 | 10.7 | 11.1 (8.8-13.4) | 1.66 (1.15-2.42) |
| Ona Ara | Peri-urban/ mixed | 914 | 65 | 15.5 | 16.0 (11.9-20.1) | 1 |
| Ibadan North-East & South-East | Urban | 17041 | 96 | 9.7 | 9.9 (7.8-11.9) | 0.63 (0.44-0.89) |
| Sangre Grande, Tunapuna/Piarco, San Juan/Laventille, Diego Martin, Chaguanas | Mixed rural/ peri-urban/ urban | 351 | 351 | 41.2 | 42.4 (37.9-46.8) | 1 |
| Arima & Port of Spain | Urban | 3030 | 111 | 120.7 | 131.6 (106.9-156.3) | 2.95 (2.27-3.84) |

**Table 10.** Rate of untreated psychosis by area of residence (<2 years duration only – grouped)

| **Local area** | **Type of area** | **Pop density**  **(per sq km)** | **New cases (2yrs)** | **Crude**  **rate*** | **Adjusted rate** (95% CI)** | **IRR** (within site)** |
| --- | --- | --- | --- | --- | --- | --- |
| Uthiramerur & Maduranthakam | Rural | 356 | 22 | 3.5 | 3.5 (2.0-5.0) | 1 |
| Chengelpettu/ Thiroporur | Peri-urban/ mixed | 737 | 54 | 6.4 | 6.6 (4.8-8.4) | 1.82 (1.11-2.98) |
| Ona Ara | Peri-urban/ mixed | 914 | 47 | 11.2 | 11.8 (8.2-15.4) | 1 |
| Ibadan North-East & South-East | Urban | 17041 | 61 | 6.2 | 6.2 (4.5-7.8) | 0.54 (0.36-0.81) |
| Sangre Grande, Tunapuna/Piarco, San Juan/Laventille, Diego Martin, Chaguanas | Mixed rural/ peri-urban/ urban | 351 | 318 | 37.3 | 38.5 (34.2-42.7) | 1 |
| Arima & Port of Spain | Urban | 3030 | 88 | 95.7 | 104.4 (82.4-126.4) | 2.55 (1.93-3.36) |

**References (supplementary material)**

Humeniuk, R., Ali, R., Babor, T.F., Farrell, M., Formigoni, M.L., Jittiwutikarn, J., De Lacerda, R.B., Ling, W., Marsden, J., Monteiro, M. and Nhiwatiwa, S., 2008. Validation of the alcohol, smoking and substance involvement screening test (ASSIST). Addiction, 103(6), pp.1039-1047.

Roberts, T., Gureje, O., Thara, R., Hutchinson, G., Cohen, A., Weiss, H.A., John, S., Pow, J.L., Donald, C., Olley, B. and Esponda, G.M., 2020. INTREPID II: protocol for a multistudy programme of research on untreated psychosis in India, Nigeria and Trinidad. BMJ open, 10(6), p.e039004.
